# Impact of myocardial scar burden on microvascular resistance reserve in patients with coronary artery disease

**DOI:** 10.1007/s00259-025-07112-6

**Published:** 2025-02-20

**Authors:** Masahiro Hoshino, Roel Hoek, Ruurt. A. Jukema, Jorge Dahdal, Pepijn van Diemen, Luuk H. G. A. Hopman, Pieter Raijmakers, Roel Driessen, Jos Twisk, Ibrahim Danad, Tsunekazu Kakuta, Juhani Knuuti, Paul Knaapen

**Affiliations:** 1 Departments of Cardiology, Amsterdam UMC, Vrije Universiteit Amsterdam, De Boelelaan 1117, Amsterdam, 1081 HV The Netherlands; 2 Department of Cardiovascular Diseases, Faculty of Medicine, Clínica Alemana de Santiago, Clínica Alemana Universidad del Desarrollo, Santiago, Chile; 3 Radiology, Nuclear Medicine & PET Research, Amsterdam UMC, Vrije Universiteit Amsterdam, Amsterdam, The Netherlands; 4 Epidemiology & Data Science, Amsterdam UMC, Vrije Universiteit Amsterdam, Amsterdam, The Netherlands; 5 Department of Cardiology, University Medical Center Utrecht, Heidelberglaan 100, Utrecht, 3584 CX The Netherlands; 6 Department of Cardiology, Tsuchiura Kyodo General Hospital, Ibaraki, Japan; 7 Turku PET Centre, Turku University Hospital and University of Turku, Turku, 20520 Finland; 8 Clinical Physiology, Nuclear Medicine and PET, Turku University Hospital and University of Turku, Turku, 20520 Finland

**Keywords:** Microvascular resistance reserve, Fractional flow reserve, [^15^O] H_2_O PET, Late gadolinium enhancement, Cardiac magnetic resonance imaging

## Abstract

**Purpose:**

The impact of myocardial scar on coronary microcirculation is not well understood. This study aims to evaluate the association between microvascular resistance reserve (MRR) and scar tissue.

**Methods:**

In this post-hoc analysis of the PACIFIC 2 trial, symptomatic patients with prior myocardial infarction (MI) and/or percutaneous coronary intervention (PCI) underwent [^15^O]H_2_O positron emission tomography (PET), cardiac magnetic resonance (CMR) imaging, and fractional flow reserve (FFR). MRR was assessed utilizing PET-derived coronary flow reserve and FFR measurements. Scar quantification was assessed by CMR late gadolinium enhancement (LGE). Vessel LGE burden was defined as the scar tissue proportion in each myocardial territory. Total LGE burden was defined as the proportion of overall scar.

**Results:**

The study included 154 patients with 397 vessels with a mean MRR of 3.56 ± 1.24. Patients with any scar tissues (LGE > 0%) exhibited a lower MRR in every myocardial territory than those without scar tissues. After adjusting for cardiovascular risk factors, either vessel LGE burden (β =-0.013, *P* = 0.006) or total LGE burden (β =-0.039, *P* = 0.002) independently predicted a reduced MRR. Compared to myocardial territories without scar tissues (LGE burdens = 0%), MRR was significantly lower in myocardial territories with vessel LGE burden = 0% + total LGE burden > 0%, and in myocardial territories with both LGE burdens > 0%.

**Conclusion:**

Scar burden was negatively associated with MRR in patients with prior MI and/or PCI. Our findings indicate that both the proportion of myocardial scar in the vascular territory and the overall myocardial scar affect the microcirculation of individual vascular territories.

**Clinical trial number:**

Not applicable.

**Supplementary Information:**

The online version contains supplementary material available at 10.1007/s00259-025-07112-6.

## Introduction

Recently, there has been a growing interest in coronary microvascular dysfunction (CMD), as it has been demonstrated to be adversely associated with prognosis [1, 2]. Therefore, the accurate evaluation of microcirculation in various conditions is becoming increasingly important.

Pathologically, it has been demonstrated that the microcirculation is damaged after myocardial infarction (MI) [3, 4]. Previous studies have demonstrated that CMD during the acute phase of MI is associated with subsequent cardiac dysfunction, adverse remodeling, and poor prognosis [5, 6]. It has been suggested that an impaired microcirculatory function may improve in the chronic phase [7]. However, there have been very few clinical studies that have clearly demonstrated the relationship between the extent of microcirculatory injury and CMD in the chronic phase in patients with a prior MI. It is well known that collaterals develop after MI, which indicates a potential compensatory mechanism after MI [4]. However, the compensatory capacity of this mechanism has not been linked to infarct size.

By evaluating the impact of scar on the microcirculation in the regions affected by myocardial injury, as well as considering the extent of myocardial injury across the entire heart, we can better understand the potential interplay between microcirculation and myocardial damage, regardless of whether the scar is transmural or non-transmural. While patchy (non-transmural) scar may retain partial blood flow, it remains unclear whether this residual perfusion is sufficient to preserve fully normal microcirculatory function in the chronic phase.

This study utilizes the microvascular reserve ratio (MRR), a novel marker proposed by De Bruyne and Pijls et al., to evaluate pure microvascular circulation without the need to account for myocardial mass or collateral circulation [8]. By integrating invasive fractional flow reserve (FFR) and positron emission tomography (PET)-derived coronary flow reserve (CFR) measurements, it is possible to assess the microcirculatory status of the myocardium accurately. As such, this study aims to elucidate the relationship between scar tissue burden, as assessed by cardiac magnetic resonance imaging (CMR), and MRR in patients with chronic coronary syndrome.

## Methods

### Patient selection

The study is a post-hoc analysis of the PACIFIC 2 study [9]. The PACIFIC 2 trial was a prospective, single-center, head-to-head comparative study from 2014 to 2020 at Amsterdam University Medical Center, location VU Medical Center in Amsterdam, the Netherlands. Symptomatic patients with a cardiac history, defined as prior MI and/or percutaneous coronary intervention (PCI), completed a two-week protocol including [^15^O]H_2_O PET and CMR imaging with late gadolinium enhancement (LGE) prior to invasive coronary angiography (ICA) coupled with routine 3-vessel invasive FFR examination. For the current study, all patients that had CMR imaging with volumetric LGE assessment were eligible for inclusion in this study.

The VUmc Medical Ethics Review Committee approved the study protocols and complied with the Declaration of Helsinki, with written informed consent was obtained from all participants.

### PET procedure

Image data sets were transferred to core laboratory (Turku University Hospital, Turku, Finland). PET scans were performed on a hybrid PET/CT equipment (Philips Gemini TF 64, Philips Healthcare, Best, The Netherlands). The scan procedure involved a dynamic six-minute scanning protocol that initiated simultaneously with an injection of 370 MBq [^15^O]H_2_O during resting and adenosine-induced hyperemic conditions (140 µg/kg/min). Low-dose CT scans allowed for attenuation correction. Parametric images of quantitative hyperemic MBF were created for each of the 17 segments of the left ventricle as per the American Heart Association model with standardized allocation of segments to the three vascular territories [10]. Images were analyzed using Carimas software (Turku PET Centre, University of Turku and Turku University Hospital, Turku, Finland). The participants were asked to abstain from caffeine or xanthine intake 24 h prior to the PET scan. Parametric MBF images were analyzed. Regional hyperemic myocardial blood flow (hMBF) was defined as mean hMBF of the entire vascular territory in the absence of a perfusion defect or as the mean hMBF of the perfusion defect (≥ 2 adjacent segments with a hMBF ≤ 2.3 ml/min/g) when present [11]. CFR was defined as the ratio of hMBF to resting MBF (rMBF).

### CMR

Images were acquired on a 1.5-T whole body MR scanner (Magnetom Avanto, Siemens Healthineers). LGE was performed using a 2-dimensional segmented inversion-recovery gradient-echo pulse sequence, following a 0.075 mmol/kg bolus of a gadolinium-based contrast agent (DOTAREM; Guerbet, Villepinte, France). If LGE was considered visually present, total infarct size (in grams) was calculated from the LGE images using the full width at half maximum method [12]. LGE was further evaluated on short-axis cardiac MRI images according to a 5-point scoring system (0–4) recommended by prior study [13]. Briefly, each myocardial segment was visually inspected from endocardium to epicardium, and the proportion of wall thickness showing hyperenhancement was estimated. A score of 0 indicated no hyperenhancement, 1 corresponded to 1–25%, 2 to 26–50%, 3 to 51–75%, and 4 to 76–100%. Segments assigned a score of 4 were defined as transmural infarctions. Infarct mass was expressed as a percentage of myocardial mass per segment according to the AHA 17-segment model excluding the apex [14]. Additionally, in accordance with the AHA model, infarct size and percentage for each vascular territory was calculated. Vessel LGE burden was defined as the scar tissue proportion in each myocardial territory of the corresponding vessel. Total LGE burden was expressed as a percentage LGE of the entire myocardial mass, considering the cumulative infarct burden across all segments. LGE analysis was performed using Circle CVI42 (version 5.13, Circle Cardiovascular Imaging, Inc, Calgary, Canada) by a researcher blinded to clinical characteristics.

### Invasive coronary angiography and physiological assessments

ICA was performed according to standard clinical protocols. Patients were instructed to refrain from the intake of xanthine or caffeine 24 h prior to the coronary angiography. All major coronary arteries (> 2 mm) were routinely interrogated by FFR, irrespective of stenosis severity and imaging results. To induce maximal coronary hyperemia, adenosine was administered intracoronary as a 150 µg bolus or intravenously (140 µg/kg/min). FFR was calculated as the ratio of mean distal intracoronary to aortic guiding pressure during hyperemia.

MRR was derived based on the framework by De Bruyne and Pijls et al. by combining invasive FFR measurements and non-invasive PET flow measurements [8]. The formula used in the current analysis is a quotient of CFR and FFR with the correction for the impact of hemodynamics, as follows:

1 $$\:MRR\:=(CFR/FFR)\:\times\:\:(Pa,\:rest/Pa,\:hyper)$$

MRR =(CFR/FFR) × (Pa, rest/Pa, hyper).

CFR indicates PET-derived coronary flow reserve, FFR indicates pressure-wire derived fractional flow reserve, and P_*a, rest*_ and P_*a, hyper*_ indicate mean aortic pressure during non-hyperemic and maximal hyperemic PET, respectively. Further details regarding the rationale for MRR are provided in the supplemental material. When there is a chronic total occlusion (CTO), the FFR of the coronary artery region cannot be accurately measured and was excluded.

### Statistical analysis

Continuous variables were presented as mean ± SD or median with IQR, based on their distribution. Categorical variables were expressed as frequencies and percentages. Vessel-specific MRR between vessels with and without vessel LGE burden was assessed using the Mann-Whitney U test. Spearman correlation was used to assess the correlations between vessel-specific MRR and LGE burden for each of the three coronary branches as well as for total LGE volume. Generalized Estimating Equations (GEE) were used to handle the correlation of multiple vessels within a single patient, ensuring accurate analysis by accounting for this clustering. To correct for cardiovascular risk factors and for multiple vessels within a patient, multivariable analyses were used to assess the relation between scar tissue and vessel-specific MRR. Due to the high correlation between vessel LGE burden and total LGE burden (*r* = 0.81, *P* < 0.001), they were analyzed separately in the multivariate analysis. To estimate the standard error of the estimate (SEE) for the relationship between LGE burden and microvascular function, we applied a log transformation log(LGE burden + 1) to accommodate the many zero values.

A 2-sided *P* < 0.05 was considered statistically significant. Statistical analyses were performed using R version 4.3.1 (R Foundation for Statistical Computing, Vienna, Austria).

## Results

**Fig. 1:**
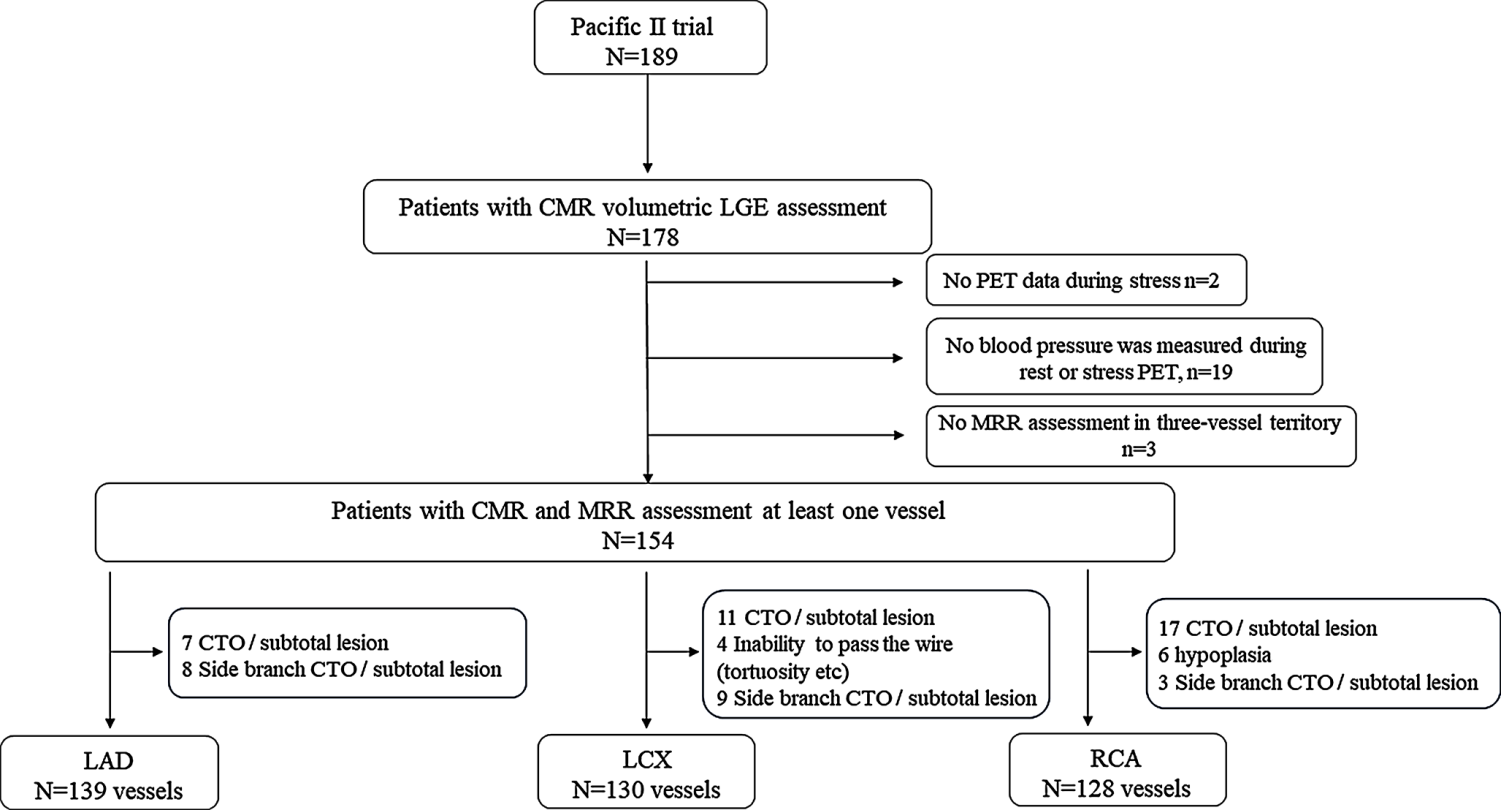
Study flow chart Flow chart of participants from PACIFIC 2 trial at Amsterdam UMC. Abbreviations: CMR, cardiac magnetic resonance; CTO, chronic total occlusion; LAD, left anterior descending artery; RCA, right coronary artery; LCX, left circumflex artery; MRR, microvascular resistance reserve; PET, positron emission tomography; LGE, late gadolinium enhancement

Among the 189 patients enrolled in the PACIFIC 2 study, 178 (94%) patients underwent CMR assessment with volumetric LGE assessment. Two patients were excluded due to the absence of stress PET scans. Nineteen were excluded due to the lack of blood pressure measurements during rest or stress PET. Three patients were excluded due to no MRR assessment in any of the three-vessel territories. Branches where FFR was not measured due to CTO, hypoplasia, severe stenosis, or inability to pass the wire were excluded. The final analysis included 154 patients with MRR assessment in at least one vessel. (397 vessels: LAD 139, LCX 130, RCA 128). (Fig. 1)

The patients’ mean age was 63.1 ± 9.0 years, and 123 (80%) were male. Of the patients, 78 (51%) had prior MI, and 138 (90%) had prior PCI. Detailed patient characteristics are described in Table 1. Vessel-specific characteristics are displayed in Table 2. Vessel LGE burden was 0% (0-3.4) for all vessels, 0% (0-1.9) for LAD, 0% (0-4.9) for RCA, and 0% (0-4.1) for LCX. Total LGE burden was 0.9% (0-5.2).

**Table 1:** Baseline characteristics

	All patients *n* = 154
**Characteristics**	
Male	123 (79.8%)
Age, years	63.1 ± 9.0
BMI, kg/m^2^	27.4 ± 4.1
Prior PCI	138 (89.6%)
Prior MI	78 (50.6%)
**Cardiovascular risk factors**	
Diabetes Mellitus	34 (22.1%)
Hypertension	95 (61.7%)
Hypercholesterolemia	103 (66.9%)
Currently smoking	22 (14.3%)
Family history of CAD	78 (50.6%)

*Abbreviations*: BMI, body mass index; PCI, percutaneous coronary intervention; MI, myocardial infarction; CAD, coronary artery disease

**Table 2:** Vessel characteristics

	All vessels *N* = 154 (397 vessels)				
	Overall	LAD	RCA	LCX	
FFR	0.90 (0.82–0.96)	0.83 (0.77–0.89)	0.93 (0.87–0.97)	0.94 (0.86–0.99)	
CFR	2.98 ± 1.24	2.73 ± 1.08	3.18 ± 1.44	3.03 ± 1.12	
MRR	3.56 ± 1.24	3.50 ± 1.16	3.68 ± 1.39	3.50 ± 1.15	
Vessel LGE burden %	0 (0-3.4)	0 (0-1.9)	0 (0-4.9)	0 (0-4.1)	
Total LGE burden %	0.9 (0-5.2)				

*Abbreviations*: FFR, fractional flow reserve; CFR, coronary flow reserve; MRR, microvascular resistance reserve; LGE, late gadolinium enhancement

### Correlation analysis: MRR, FFR, and LGE

**Fig. 2:**
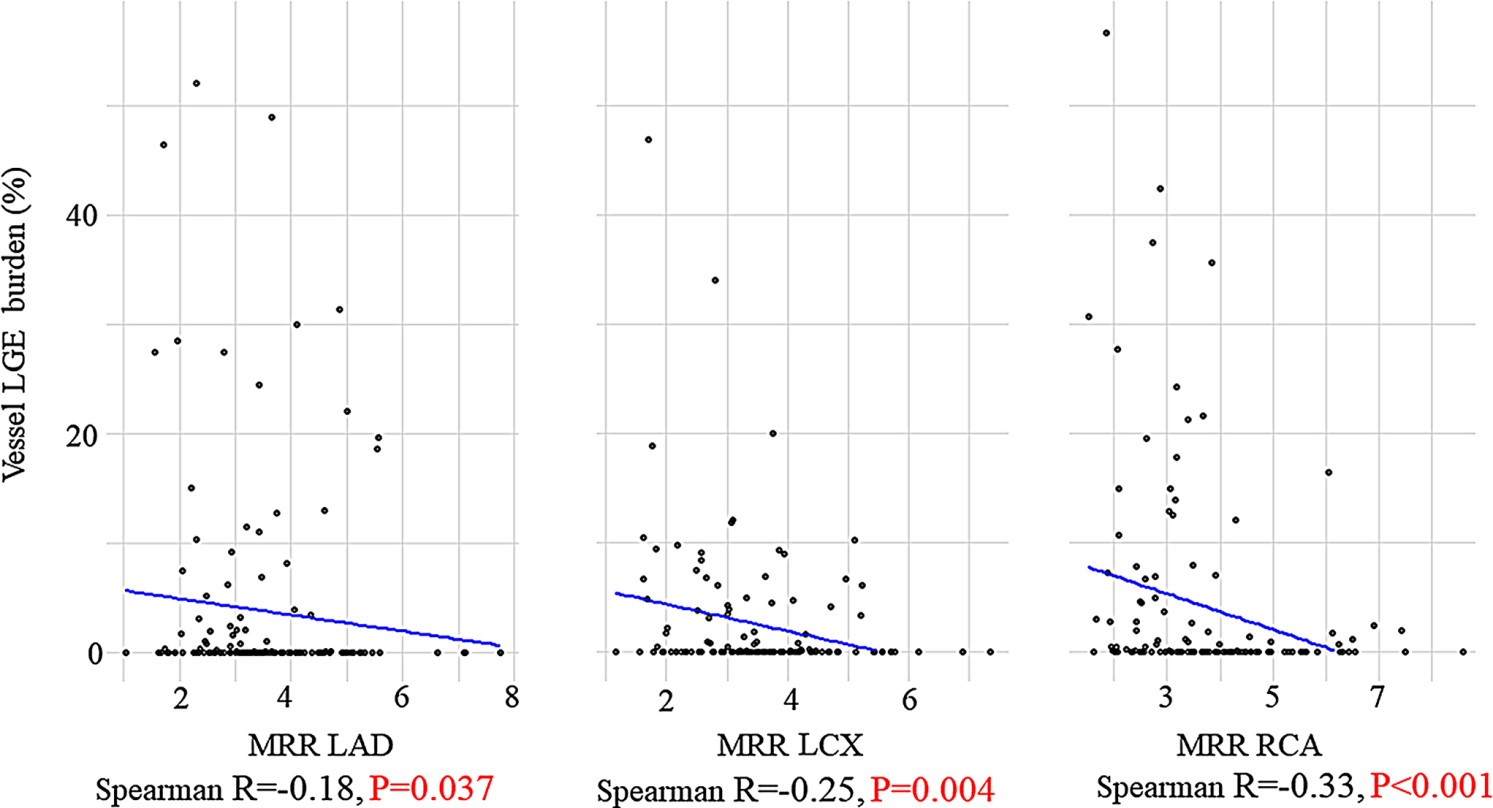
Association between vessel LGE burden and vessel-specific MRR. Correlation Analysis between vessel LGE burden and vessel-specific MRR. Abbreviations are in Fig. 1

**Fig. 3:**
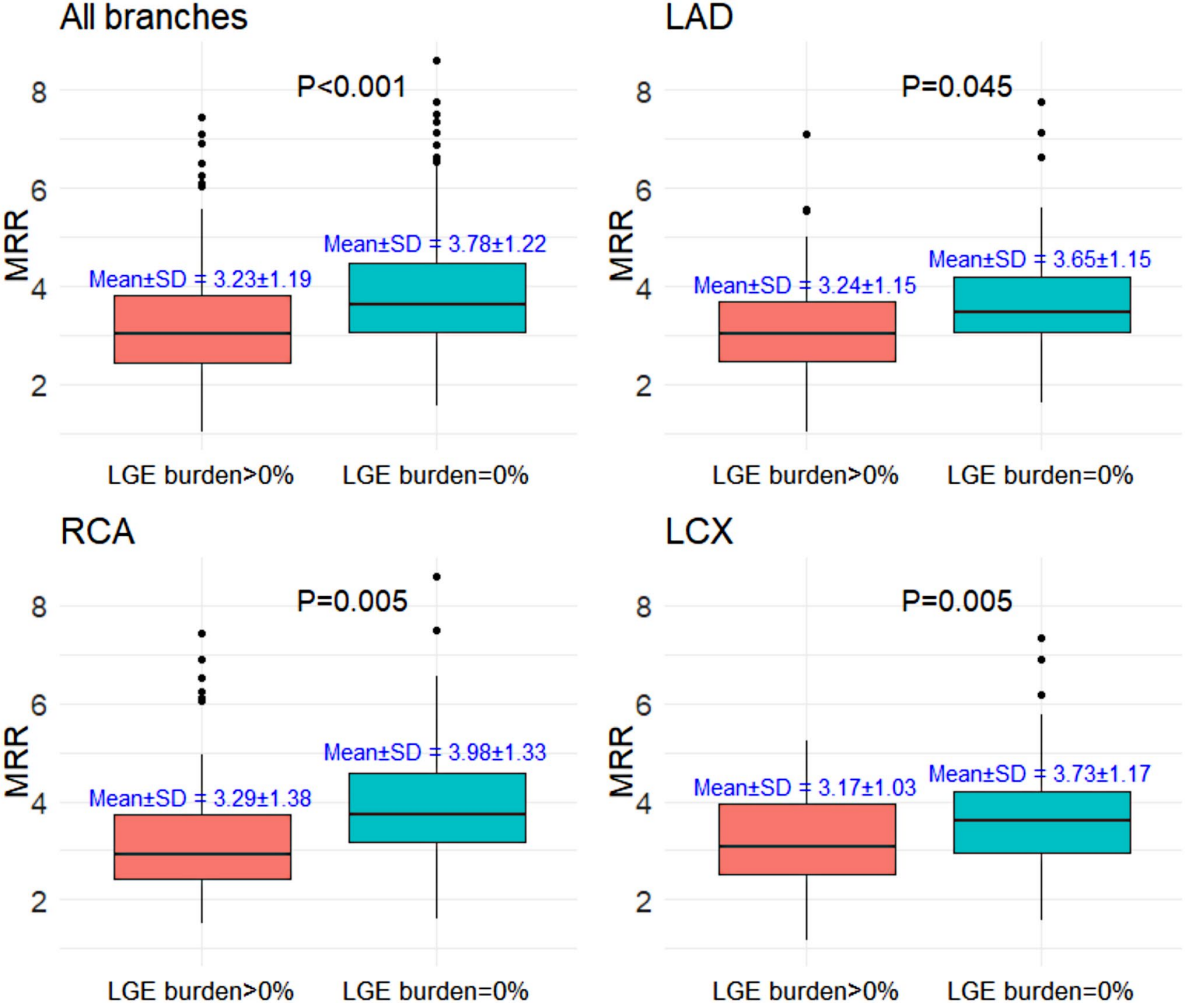
Comparison of MRR based on the presence or absence of vessel LGE burden. The top-left panel shows all branches combined, the top-right panel shows LAD, the bottom-left panel RCA, and the bottom-right panel LCX. Each panel compares territories with LGE (> 0%) against those without LGE (0%).For each coronary territory, the MRR values are compared between myocardial regions with the presence of scar tissue (vessel LGE burden > 0) and those without scar tissue (vessel LGE burden = 0). Abbreviations are in Fig. 1

Vessel LGE burden showed a negative Spearman correlation with LAD-MRR (Spearman *r*=-0.18, *P* = 0.037), LCX-MRR (Spearman *r*=-0.25, *P* = 0.004), and RCA-MRR (Spearman *r*=-0.33, *P* < 0.001) (Fig. 2). Vessels in myocardial territories with any scar tissue (vessel LGE burden > 0) had a significantly lower MRR than myocardial territories without scar tissue (Fig. 3). Similarly, CFR was significantly lower in coronary territories with LGE than in those without LGE (Supplemental Fig. 1). On a per-patient basis using total LGE burden, a negative correlation with LAD-MRR (Spearman *r*=-0.21, *P* = 0.011), LCX-MRR (Spearman *r*=-0.18, *P* = 0.041), and RCA-MRR (Spearman *r*=-0.30, *P* < 0.001) was observed (Supplemental Fig. 2). FFR was not significantly correlated to vessel-specific MRR and vessel-specific LGE burden (Supplemental Fig. 3 and Supplemental Fig. 4). In a sensitivity analysis excluding vessels with MRR > 6.0 (Supplemental Fig. 5), the correlation between MRR and LGE burden remained significant.

### Impact of LGE burden on MRR

Linear regression analysis indicated that in a univariable analysis, diabetes (β=-0.546, *P* = 0.005), smoking status (β=-0.626, *P* < 0.001), vessel LGE burden (β=-0.014, *P* = 0.004), and total LGE burden (β=-0.041, *P* = 0.005) were associated with vessel-specific MRR. (Table 3). After adjusting for cardiovascular risk factors (age, diabetes, smoking status, and hypertension), when either vessel LGE burden (β = -0.013, *P* = 0.006) or total LGE burden (β = -0.039, *P* = 0.002) was included in the model, each independently predicted reduced MRR. Additionally, when comparing the three groups, MRR was significantly lower in the following groups compared to myocardial territories with both vessel LGE burden and total LGE burden equal to zero: (1) myocardial territories with vessel LGE burden equal to zero and total LGE burden greater than zero (β = -0.419, *P* = 0.026). (2) myocardial territories with both vessel LGE burden and total LGE burden greater than zero (β = -0.423, *P* = 0.015). In a subgroup analysis (Supplemental Fig. 6), CFR and MRR decreased stepwise across territories with no LGE, subendocardial non-transmural LGE, and transmural LGE.

**Table 3:** Results of the GEE analyses to analyze the relationship between scar tissue and vessel-specific MRR

	Univariable analysis		Multivariable analysis 1			Multivariable analysis 2		Multivariable analysis 3	
	β	P value	β	P value		β	P value	β	P value
Age	-0.016	0.087	-0.021	0.099		-0.022	0.014	-0.021	0.027
Male	-0.029	0.900							
BMI	-0.035	0.120							
Diabetes Mellitus	-0.546	0.005	-0.448	0.019		-0.412	0.034	-0.434	0.030
Hypercholesterolemia	-0.225	0.260							
Hypertension	-0.343	< 0.001	-0.292	0.099	-0.329		0.062	-0.312	0.070
Current smoker	-0.626	< 0.001	-0.668	< 0.001	-0.652		< 0.001	-0.636	0.001
Vessel LGE burden %	-0.014	0.004	-0.013	0.006					
Total LGE burden %	-0.041	0.005			-0.039		0.002		
Group according to scar pattern									
① Both vessel LGE burden and total LGE burden = 0 (*N* = 183)	Reference	-						Reference	-
② Vessel LGE burden = 0 and total LGE burden > 0 (*N* = 55)	-0.489	0.015						-0.419	0.026
③ Both vessel scar burden and total scar burden > 0 (*N* = 159)	-0.478	0.011						-0.423	0.015

*Abbreviations*: BMI, body mass index; MRR, microvascular resistance reserve; LGE, late gadolinium enhancement

## Discussion

This sub-study from the Pacific-2 trial provides important insights into the relationship between myocardial scar burden and MRR in patients with a history of CAD. We found that the presence of scar tissue, quantified by LGE on CMR imaging, is associated with a reduced MRR in all coronary branches. This negative association persisted even after adjusting for cardiovascular risk factors, suggesting that both vessel-specific and total myocardial scar burdens are independent predictors of microvascular dysfunction.

### Temporal changes and challenges in assessing microcirculation after myocardial infarction

Ischemic cardiomyopathy (ICM) arises from irreversible loss of viable myocardial mass and dysfunctional but viable myocardium due to chronically reduced blood flow. While acute phase dysfunction has been linked to physiological markers and prognosis, there are no reports suggesting a direct relationship between myocardial damage and microcirculation in chronic phase patients [15, 16]. There have been reports of microcirculatory dysfunction improving over time following acute myocardial infarction [7]. From a pathological perspective, microvascular damage after MI is characterized by the accumulation of fibrin thrombi and necrotic myocardial cell fragments in the microcirculation [3]. Neovascularization occurs as part of the healing process. However, as viable myocardial mass decreases, a relative balance between blood supply and demand is achieved in the chronic phase, tending to improve microvascular function itself. Therefore, while microcirculation deteriorates in the acute phase of MI, its impact on chronic phase microcirculation is uncertain. Moreover, as viable myocardium decreases, IMR tends to increase due to its dependence on myocardial mass [17]. Moreover, since IMR is often affected by collateral flow, which frequently occurs after MI, evaluating the state of microcirculation after MI is challenging. These factors might explain why only about one-third of the microcirculatory dysfunction defined by IMR corresponds with the assessment of myocardial damage measured by CMR as reported in previous studies [6]. In this regard, MRR is considered more appropriate for assessing microcirculation after MI because it is not affected by collateral flow or myocardial mass [8, 18].

### Relationship between scar tissue and MRR

Since FFR increases with a decrease in viable myocardium [19], only MRR, a pure marker of microvascular function independent of myocardial mass and FFR [8], could accurately evaluate the relationship between chronic myocardial damage and microcirculation. In this study, no significant relationship was observed between FFR and myocardial damage, suggesting that the relationship between decreased MRR and myocardial damage purely reflects the outcomes of microcirculation and myocardial damage. Although neovascularization occurs in response to myocardial damage, the microvascular function is likely to decline even in the chronic phase due to the influence of scarred areas [3, 4]. Interestingly, this effect remains significant even after considering factors known to impact microcirculation, such as age, hypertension, diabetes, and smoking status [20].

Our data suggest that myocardial scar may have a global influence on the coronary microcirculation, rather than confining its effects solely to the infarcted region. In particular, we found that territories without localized LGE (0% LGE) also showed reduced MRR in patients with a larger total scar burden, suggesting a broader, whole-heart impact of myocardial damage. Although the precise mechanism remains to be determined, these findings underscore that microvascular dysfunction can extend beyond the site of local scar and should be interpreted in the context of overall myocardial health. Collateral vessels, forming small arterial connections that expand to provide alternative blood supply, play a protective role in myocardial ischemia and left ventricular dysfunction [4]. Well-developed collateral vessels improve patient prognosis, reduce mortality risk, maintain cardiac function, and limit infarct size during myocardial infarction [21]. Additionally, the presence of scar tissue elsewhere in the heart, indicative of more advanced CAD, can lead to reduced MRR even in territories without localized scar tissue. This highlights that microvascular dysfunction is more prevalent in patients with more advanced disease. Therefore, it is necessary to consider that microcirculation after myocardial infarction may deteriorate not only in the infarcted area but also globally.

Moreover, we performed subgroup analyses differentiating transmural from non-transmural scars and found a stepwise decrease in MRR from no- to subendocardial non-transmural- to transmural scar. Although fully transmural scar constituted only about 7% of all territories, their presence was associated with even lower MRR, suggesting that more extensive infarction may exacerbate microvascular dysfunction. Future studies with larger cohorts or a greater burden of transmural scar could help confirm these findings. In our analysis, the correlation between vessel LGE burden and MRR was modest, with a relatively large scatter (SEE = 1.06). Although this likely reflects the multifactorial nature of microvascular function and inherent measurement variability, LGE burden remained an independent predictor of MRR even after adjusting for confounders. Moreover, our findings remained significant in a sensitivity analysis excluding vessels with MRR > 6.0, supporting a robust—albeit not extremely strong—relationship between myocardial scarring and microvascular function. Lastly, CFR was also lower in LGE-positive territories. Considering that most vessels in our cohort had an FFR > 0.80, this finding suggests that CFR may capture microvascular impairments similar to those reflected by MRR under these conditions.

### Clinical implication

Previous studies have evaluated MRR regardless of a history of MI. However, it is important to recognize that MRR values tend to decrease in patients with severe scar as defined by LGE. This decline in MRR may be indicative of microvascular dysfunction that persists beyond the acute phase of the infarction, reflecting ongoing challenges in the microcirculatory system.

### Limitation

Some limitations should be acknowledged. Firstly, this was a cross-sectional study. Secondly, this study population is restricted to patients with a history of CAD, making it inapplicable to patients without such a history. Thirdly, this study did not entail endothelial function tests for assessing arteriolar dysregulation in the coronary microcirculation. Fourthly, the vessel territories used are the general territories in the AHA model, but can differ between patients according to their coronary artery anatomy. Fifthly, although patients with prior MI were included, the regional extensive scar was scarce, which may underestimate its impact. In our study, even small scar areas are associated with lower MRR. Sixthly, the tracer of [^15^O]H_2_O has the unique ability to be linearly related to myocardial blood flow, primarily in viable myocardium [22]. Therefore the influence of nonviable tissue (i.e. scar) could be underestimated. Seventhly, our study did not obtain invasive flow and resistance values, as would typically be acquired for invasive measurement of MRR. Eighthly, although 50.6% (78/154) of our study population had a prior myocardial infarction and 89.6% (138/154) had a history of PCI, we cannot fully exclude non-ischemic causes (e.g., myocarditis or infiltrative processes) for certain LGE findings. Additionally, we cannot fully exclude non-ischemic causes for some LGE findings (e.g., inflammatory or infiltrative processes). While 67 patients (43.5%) showed LGE of ≥ 50% wall thickness, suggesting significant injury [13], many others had minimal or no LGE, which limited our ability to assess the impact of large infarcts. We also lacked detailed information on ischemia duration and symptom status, preventing correlation of microvascular dysfunction with long-standing perfusion deficits or clinical presentations. Future prospective research with more extensive imaging and clinical data is needed to address these constraints. Moreover, although all patients underwent imaging at least three months after their last known ischemic event—likely enough time for microcirculatory adaptation to stabilize [7]—we did not record the precise interval for each individual. Since microcirculatory recovery and remodeling can continue to evolve, this lack of uniform timing data may confound the interpretation of MRR and LGE in chronic versus late subacute phases. Lastly, we defined any LGE > 0% as “scar,” which may include minimal LGE (e.g., 0.1%) as well. We therefore performed a sensitivity analysis applying a 1% cutoff, finding that our results remained overall consistent (Supplemental Fig. 7). This highlights that small amounts of LGE could still be relevant to microvascular dysfunction, but we cannot exclude a degree of measurement or biological variability at such low levels.

## Conclusion

This study provides important insights into the potential negative impact of myocardial scar burden on MRR in patients with a history of prior MI and/or PCI. Our findings suggest that both the proportion of myocardial damage within specific vascular territories as well as the overall percentage of myocardial scar adversely affect the MRR. These results stress the possible role that myocardial scarring plays in microvascular health and emphasize the necessity for comprehensive cardiovascular assessment in these patients.

## Electronic supplementary material

Below is the link to the electronic supplementary material.

Supplementary Material 1

Supplementary Material 2

Supplementary Material 3

Supplementary Material 4

Supplementary Material 5

Supplementary Material 6

Supplementary Material 7

Supplementary Material 8

## Data Availability

The datasets analyzed during the current study are available from the corresponding author upon reasonable request.

## Abbreviations

CFR: Coronary Flow Reserve
CMD: Coronary Microcirculatory Dysfunction
CT: Computerized Tomography
FFR: Fractional Flow Reserve
HMBF: Hyperemic Myocardial Blood Flow
MRR: Microvascular Resistance Reserve
PET: Positron Emission Tomography
