# Supplementary figures and images for: Impact of myocardial scar burden on microvascular resistance reserve in patients with coronary artery disease
Source: Eur J Nucl Med Mol Imaging. 2025 Feb 20;52(9):3312–20. doi: 10.1007/s00259-025-07112-6 (PMC12222363; doi:10.1007/s00259-025-07112-6)

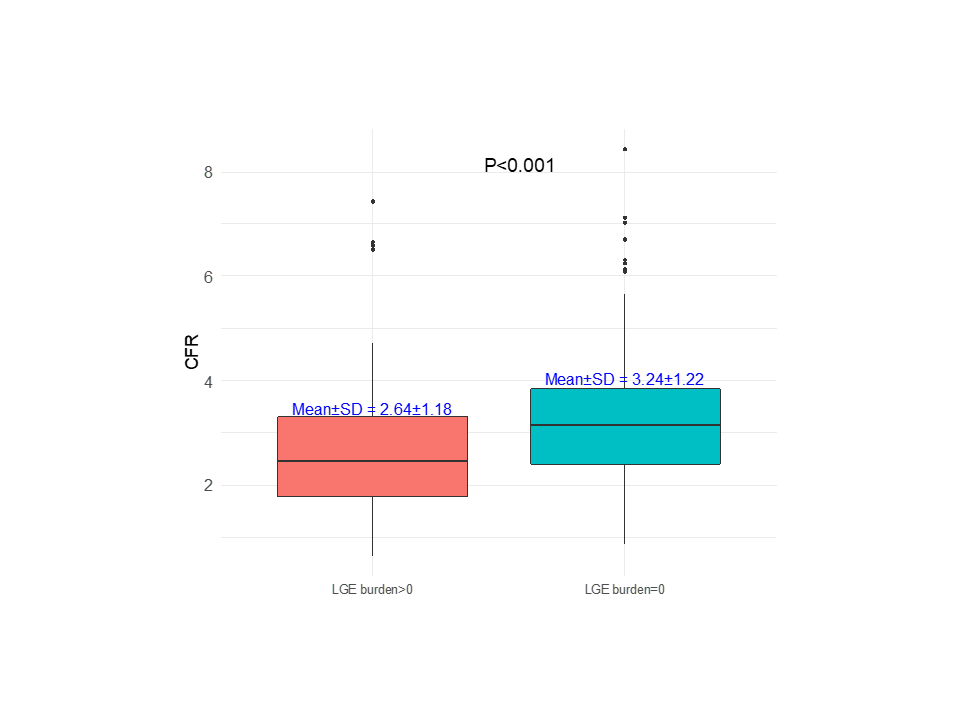

Supplement: Supplementary file 1 — Supplementary Material 1 [file 259_2025_7112_MOESM1_ESM.tif]

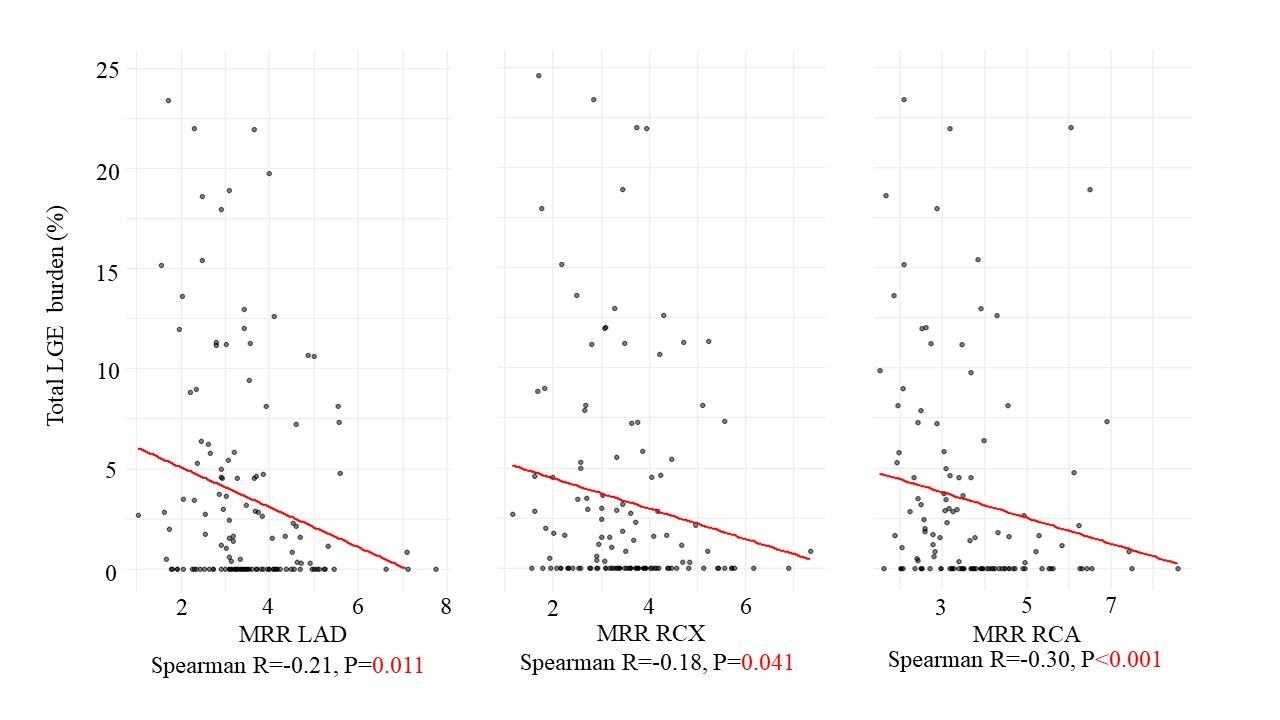

Supplement: Supplementary file 2 — Supplementary Material 2 [file 259_2025_7112_MOESM2_ESM.tif]

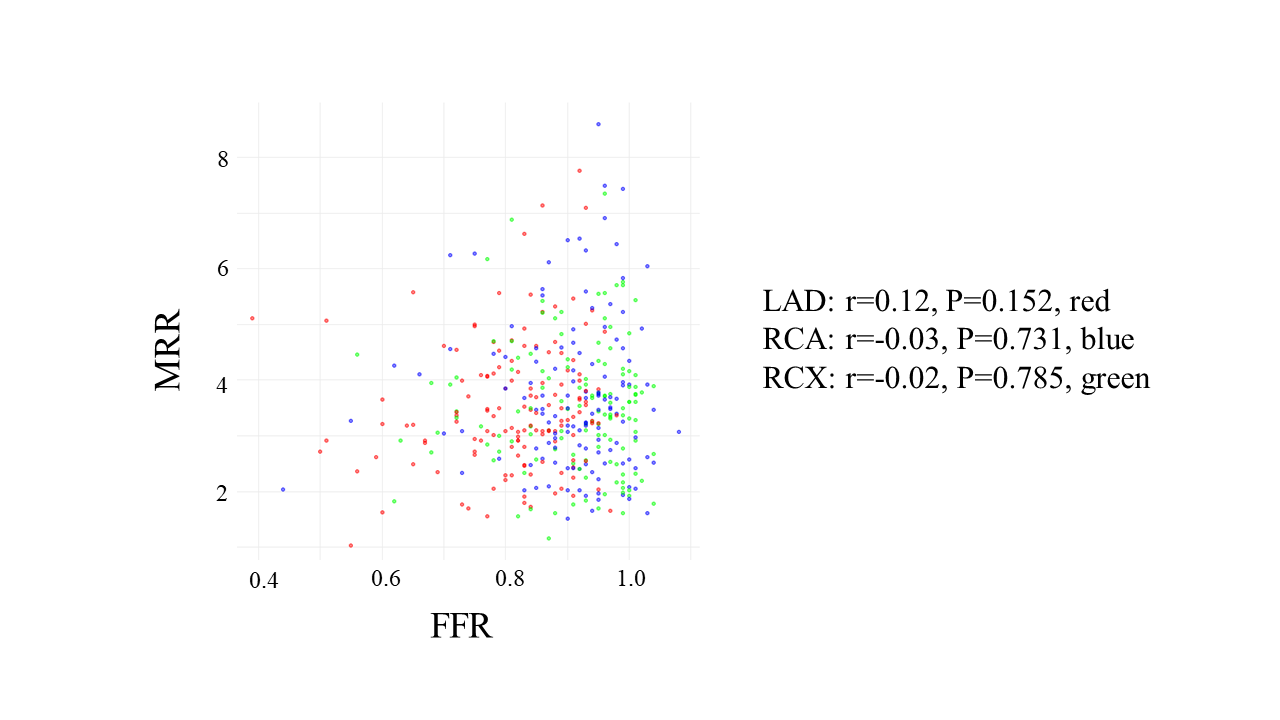

Supplement: Supplementary file 3 — Supplementary Material 3 [file 259_2025_7112_MOESM3_ESM.tif]

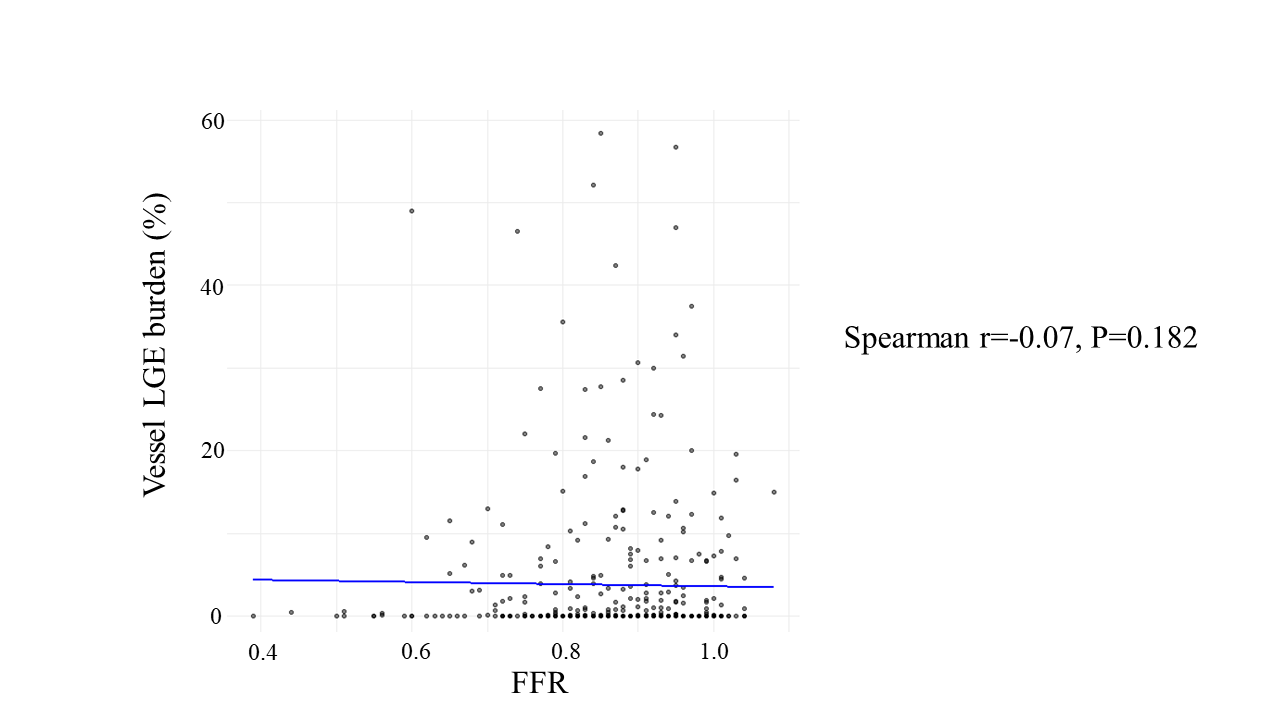

Supplement: Supplementary file 4 — Supplementary Material 4 [file 259_2025_7112_MOESM4_ESM.tif]

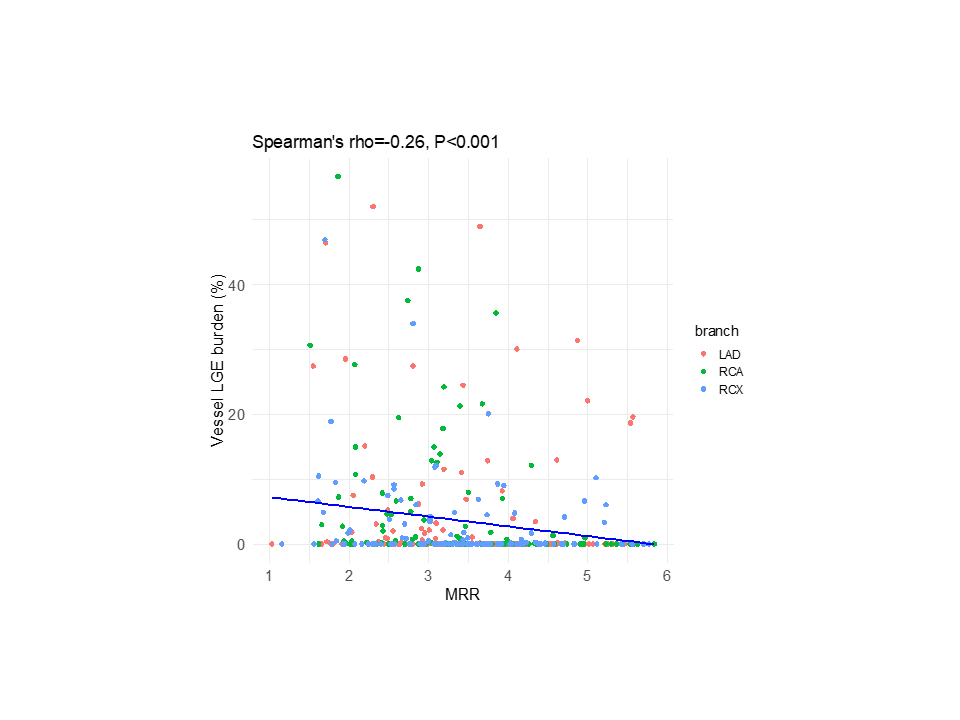

Supplement: Supplementary file 5 — Supplementary Material 5 [file 259_2025_7112_MOESM5_ESM.tif]

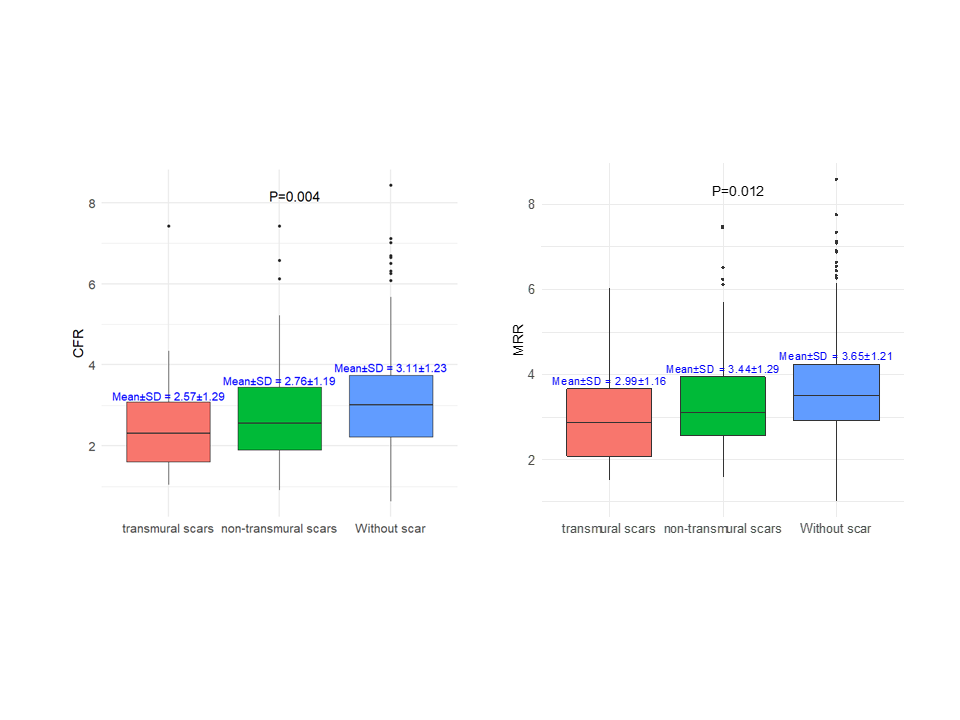

Supplement: Supplementary file 6 — Supplementary Material 6 [file 259_2025_7112_MOESM6_ESM.tif]

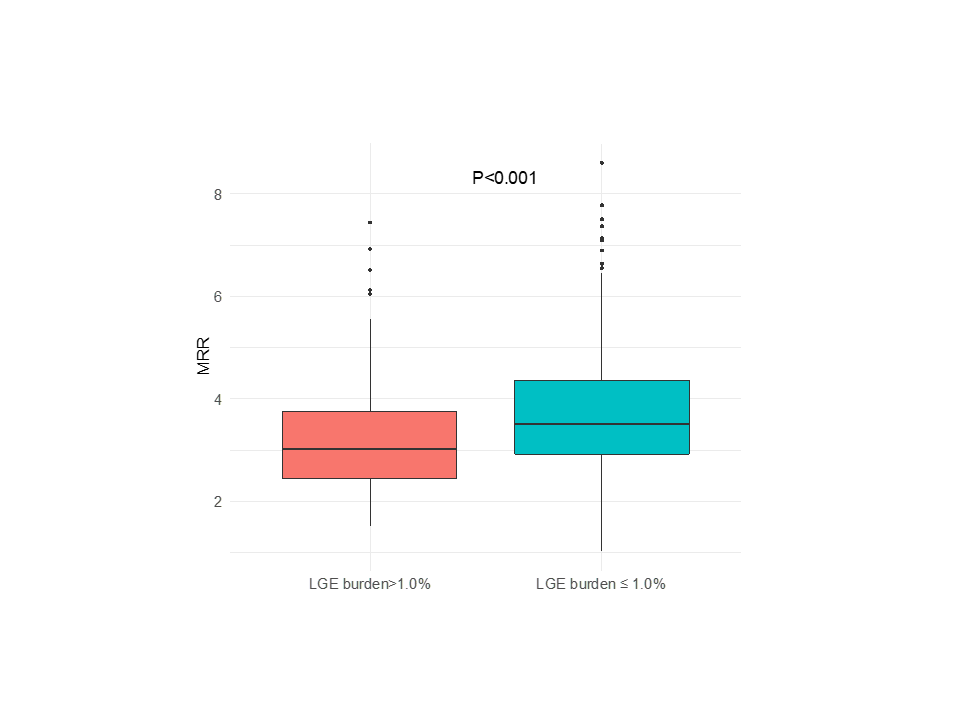

Supplement: Supplementary file 7 — Supplementary Material 7 [file 259_2025_7112_MOESM7_ESM.tif]
