# Supplementary material for: Impact of myocardial scar burden on microvascular resistance reserve in patients with coronary artery disease
Source: Eur J Nucl Med Mol Imaging. 2025 Feb 20;52(9):3312–20. doi: 10.1007/s00259-025-07112-6 (PMC12222363; doi:10.1007/s00259-025-07112-6)
Supplement: Supplementary file 8 — Supplementary Material 8 [file 259_2025_7112_MOESM8_ESM.docx]

**Supplemental Material:** Additional Details on the MRR Methodology

Mean Arterial Pressure Rather Than Diastolic Pressure
MRR follows the same convention as invasive fractional flow reserve (FFR), which uses mean distal coronary pressure and mean aortic pressure under hyperemia rather than diastolic pressure alone. Although the majority of coronary blood flow occurs in diastole, clinical pressure-wire measurements employ mean pressures to represent the entire cardiac cycle. This alignment allows for straightforward integration of invasive FFR and non-invasive coronary flow reserve (CFR) measurements.

Accuracy in the Presence of Significant Scar
Myocardial flow reserve (MFR) or CFR) can underestimate microvascular function in regions with extensive scarring, because nonviable myocardium does not contribute to flow demand. By incorporating FFR, MRR reduces the confounding impact of epicardial stenosis on flow measurements. Although MFR alone might be less accurate in territories with large scar, MRR can still detect microvascular impairment by normalizing for the epicardial contribution.

Minimal Influence of Collateral Flow and Myocardial Mass
MRR integrates CFR with invasive FFR in a way that “factors out” the epicardial component, thus highlighting microvascular physiology. Prior reports [1] suggest that MRR is minimally influenced by collateral flow because it relies on ratios of hyperemic to basal flow combined with FFR and a mean aortic pressure correction. Whereas IMR can rise when viable myocardium is reduced, MRR focuses on hyperemic-versus-resting flow ratios at the tissue level, lessening its dependence on absolute myocardial mass.

**Reference**

1. De Bruyne B, Pijls NHJ, Gallinoro E, Candreva A, Fournier S, Keulards DCJ, Sonck J, Van't Veer M, Barbato E, Bartunek J *et al*: Microvascular Resistance Reserve for Assessment of Coronary Microvascular Function: JACC Technology Corner. *J Am Coll Cardiol* 2021, 78(15):1541-1549.
